# Durable outcomes with manageable safety leading to prolonged survival with tagraxofusp for treatment-naïve patients with blastic plasmacytoid dendritic cell neoplasm: real world results from a European Named Patient Program

**DOI:** 10.1007/s00277-025-06493-w

**Published:** 2025-08-02

**Authors:** Marco Herling, Emanuele Angelucci, Tobias Matthieu Benoit, Giulia Rivoli, Antonio Curti, Katharina S. Götze, Stefan Wirths, Dimoula Erakli, Michael Zuurman, Eric Deconinck

**Affiliations:** 1 Department of Hematology, Cell Therapy, Hemostaseology, and Infectious Diseases, University of Leipzig and Cancer Center Central Germany (CCCG), Leipzig- Jena, Germany; 2 Hematology and Cellular Therapy Unit, IRCCS Ospedale Policlinico San Martino, Genova, Italy; 3 Department of Medical Oncology and Hematology, University Hospital Zurich, Zurich, Switzerland; 4 IRCCS Azienda Ospedaliero- Universitaria di Bologna, Istituto di Ematologia “Seragnoli”, Bologna, Italy; 5 Klinik und Poliklinik für Innere Medizin III, Hämatologie/Onkologie, TUM School of Medicine and Health, Dept. Clinical Medicine, München, Germany; 6 Department of Internal Medicine 2, Hematology, Oncology, Clinical Immunology and Rheumatology, University Hospital Tuebingen, Tuebingen, Germany; 7 Aixial Group, West Sussex, UK; 8 Menarini Group, Machelen, Belgium; 9 CHU Besançon Hematology, Université Marie et Louis Pasteur, UMR Right Inserm, Besançon, France

**Keywords:** Acute myeloid malignancies, Biological therapy, BPDCN, CD123, Tagraxofusp

## Abstract

Blastic plasmacytoid dendritic cell neoplasm (BPDCN) is an aggressive, poor-prognostic, and CD123-overexpressing orphan hematologic malignancy for which tagraxofusp, a CD123-targeted fusion protein, is the only approved drug. In this retrospective study, safety and efficacy of tagraxofusp were assessed through real-world clinical practice data, collected in patients with BPDCN who received tagraxofusp via a European Named Patient Program (2019–2024). Twenty-six adults with treatment-naïve BPDCN received 12 µg/kg tagraxofusp intravenously daily on days 1–5 (or by day 10) of a 21-day cycle. Primary endpoints were complete response (CR) rates and incidence/severity of capillary leak syndrome (CLS). Secondary endpoints included hematopoietic stem cell transplantation (HSCT), overall survival (OS), and safety. At a median follow-up of 13.5 months, the overall response rate (ORR) was 90% (65% CR); the median duration of response (DOR) was 10.3 months. In 12 evaluable patients who were bridged to HSCT, the pre-transplant ORR was 92% (75% CR), with 14.7 months median DOR. Median OS was 20.2 months (95% CI 10.2–not estimable) in the overall population and 37.0 months (95% CI 10.7–not estimable) in patients bridged to HSCT. CLS events were diagnosed in 13 patients (50%); 68% occurred in cycle 1; 54% were grade 2, and 46% grade 3/4. Treatment-related non-hematologic grade 3/4 adverse events (AEs) or serious AEs were reported in 12 patients (46%); 9 patients (35%) had treatment-related grade 3/4 hematologic AEs. These real-world findings showed no new safety signals and confirmed that tagraxofusp is the first-line treatment of choice for most patients with BPDCN.

**Supplementary Information:**

The online version contains supplementary material available at 10.1007/s00277-025-06493-w.

## Introduction

Blastic plasmacytoid dendritic cell neoplasm (BPDCN) is a rare and aggressive orphan hematologic malignancy characterized by clonal expansion of plasmacytoid dendritic tumor cells with a distinct immunophenotype of overexpression of CD123 and expression of other markers (e.g., CD4, CD56, CD303, TCL1) [1–5]. Tagraxofusp is a first-in-class CD123-directed therapy comprised of a recombinant human IL-3 protein fused to a truncated diphtheria toxin payload [6] and is the only drug approved in the United States and the European Union to treat patients with BPDCN [7–10]. Tagraxofusp was approved based on a multicenter, phase 1/2 study (NCT02113982), the largest prospective BPDCN trial to date and with exemplary prespecified multisystem response criteria/endpoints [7, 8, 11]. In treatment-naïve patients enrolled in the pivotal study, tagraxofusp demonstrated a 75% overall response rate (ORR: 57% complete response [CR]/complete clinical response [CRc]) and a 24.9-month median duration of CR/CRc), and with 51% of patients bridged to hematopoietic stem cell transplantation (HSCT) [7, 8]. Tagraxofusp had a manageable safety profile, with capillary leak syndrome (CLS) characterized by hypoalbuminemia, edema, weight gain, and hypotension, as the most serious treatment-associated adverse event (AE).

Historically, without approved therapies, BPDCN was difficult to treat, with chemotherapies used for other hematologic malignancies defining the treatment armamentarium [12]. Although the rates of complete remission with intensive polychemotherapy regimens ranged from 41 to 55%, they were usually short-lived, with most relapses observed within the first year [7, 13]. Polychemotherapies for acute leukemias are often especially not suitable for the (predominantly elderly) BPDCN patients, due to associated toxicities and early death [7, 13–15]. Compounding the challenges of chemotherapy, interpretation and application of available evidence is marred by retrospective study designs and lack of prespecified or multisystem response criteria/endpoints. Furthermore, HSCT (mostly allogeneic), the only potential curative therapy for adult BPDCN after successful induction, was not feasible in most patients due to older age, unfitness, and comorbidities [11, 12]. As such, an unmet medical need arose for novel, well-tolerated targeted agents for the treatment of BPDCN to offer durable responses in patients that are not eligible for HSCT or to enable successful bridging to HSCT with a strategy that is less toxic than polychemotherapy, and hence applicable to a larger population of patients [16].

Real-world observational data can provide important complementary insights into the safety and efficacy of new treatments and offer additional evidence in support of clinical trials. Since tagraxofusp is the only approved standard of care for BPDCN, we sought to investigate the outcome of treatment with tagraxofusp in Europe.

BPDCN patients in Europe were able to receive tagraxofusp through a Named Patient Program (NPP) ahead of commercial availability, which offered a unique opportunity to retrospectively collect outcomes in a real-world setting. We performed an independent chart-review analysis of patients treated with tagraxofusp in participating centers in Europe to help guide treatment decisions for patients with BPDCN to improve outcome and to complement the demonstrated effectiveness of tagraxofusp in the pivotal clinical study [7, 8]. To provide clinicians with valuable information to ensure safe and effective clinical use of tagraxofusp in daily practice, we report findings from the treatment-naïve population in this real-world study.

## Methods

### Study design

A non-interventional, retrospective, observational, multicenter, single-arm study was conducted from 2019 to 2024 in 49 sites across six countries in Europe: Austria, France, Germany, Italy, Spain, and Switzerland. Patients ≥ 18 years of age, with a confirmed diagnosis of BPDCN as per WHO criteria [5, 17] with immunophenotyping using established panels inclusive of lineage-specific markers (i.e., myeloid, monocytic, T-/B-cell) as well as CD123, CD4, and CD56, were enrolled. Treatment-naïve adults were included in this analysis.

Treatment with tagraxofusp in the NPP was at the discretion of the treating physician, and in line with the EU Summary of Product Characteristics [10]. Tagraxofusp 12 µg/kg was administered as an intravenous infusion once daily on days 1–5 (with a 10-day treatment window allowed for the five doses should dose interruption be needed) of a 21-day cycle. Hospitalization was required for the first cycle, with subsequent cycles allowed to be administered in the outpatient setting. All members of the healthcare team—physicians, nurses, pharmacists—involved with tagraxofusp patient care received training on CLS assessment and monitoring as well as clinical CLS management guidelines which included observing the patient for at least 24 hours after the last infusion in cycle 1 and at least 4 h after each infusion in subsequent cycles.

All patients identified with BPDCN and treated with tagraxofusp could be included; there were no formal exclusion criteria. Participation was approved by the local independent ethics committee or institutional review board as needed. The central ethics committee approval was provided by the sponsor, the University of Leipzig (288/22-ek).

### Endpoints

Response criteria developed in the pivotal trial [7] were utilized as follows: measurement of cutaneous manifestations by Modified Severity Weighted Assessment Tool and skin biopsy; assessment of bone marrow and peripheral blood by standard criteria for acute myeloid leukemia; and evaluation of lymph nodes and viscera as per standard criteria based on computed tomography. The objective of the study was to determine the response rate and the safety of tagraxofusp in a real-world setting. The ORR was based on the best overall response achieved (CR, CR with incomplete hematologic recovery [CRi], or partial response [PR]) at any time in patients with at least one documented tumor response assessment. The CR rate was defined as a best overall response of CR or CRi. Additional secondary objectives included rate of patients bridged to HSCT, progression-free survival (PFS), overall survival (OS), duration of response (DOR), time to response, and hematologic and non-hematologic safety, including separately captured AEs of special interest (e.g., CLS).

### Statistical methods

Given the real-world, retrospective nature of the study, there were no predefined visits, with data collected as available per routine schedule at each site. The primary analysis set included all patients who fulfilled the inclusion criteria (established diagnosis of BPDCN and treatment with tagraxofusp); the current analysis included the treatment-naïve adult population. All analyses were descriptive; no statistically powered endpoints were defined.

Tumor response, evaluation of disease, and safety were assessed by the investigators. Best overall response (BOR) was defined as the best response obtained during the tagraxofusp treatment period in the order of CR/CRi/PR/stable disease (SD)/progressive disease (PD)/unknown. DOR was defined as the period from initial PR, CRi or CR until first documented PD or death from any cause, including post-transplant remission in patients successfully bridged to HSCT. PFS was defined as the time from first tagraxofusp dose until first documented PD or death from any cause, whichever occurred first. OS was defined as the time from first tagraxofusp treatment to death. Patients with no documented progression or death were censored at the last patient status date.

Incidence of grade 1 to 4 hematologic AEs and grade 3 or 4 non-hematologic AEs and non-hematologic serious adverse events (SAEs) were recorded by system organ class and preferred terminology. Death was recorded, with reported cause as PD, other concurrent disease, infectious complication, or unknown. CLS events were recorded separately and included associated symptoms, average duration, grade (1, 2, 3, or 4), action taken with tagraxofusp (if applicable), and CLS management. Time to occurrence of a CLS event was calculated as CLS start date minus the tagraxofusp start date of the cycle in which the CLS event occurred. Maximum grade of CLS was determined in patients with more than one CLS event. Liver-related toxicities were aggregated by combining preferred terms aspartate aminotransferase increase, alanine aminotransferase increase, hepatic cytolysis, hypertransaminasemia, and hepatotoxicity. Myelosuppression-related AEs were assessed by combining preferred terms into “cytopenia.” Cytopenia was defined as all events reported under the following preferred terms: neutropenia, neutropenic sepsis, neutrophil count decreased, febrile neutropenia, anemia, hemoglobin decreased, hemolytic anemia, thrombocytopenia, and decreased platelet count. The aggregated term of “neutropenia” included the preferred terms of neutropenia, neutropenic sepsis, neutrophil count decreased, and febrile neutropenia. The aggregated term of “anemia” included the preferred terms of anemia, hemoglobin decreased, and hemolytic anemia. The aggregated term of “thrombocytopenia” included the preferred terms of thrombocytopenia and decreased platelet count. Rates of treatment-related hematologic aggregate term AEs per cycle were determined based on at-risk patients (i.e., those receiving treatment at each cycle).

HSCT data collected included disease status prior to and after HSCT, type of HSCT, incidence of graft versus host disease (GvHD), sinusoidal obstruction syndrome (SOS, formerly veno-occlusive disease [VOD] of the liver), time to HSCT since first and last tagraxofusp dose, and BPDCN diagnosis.

The 95% confidence interval (CI) for CR and CRi was calculated using the Clopper-Pearson Exact method, and the 95% CI for CLS events was calculated using Poisson Exact method. Analysis of DOR, PFS, and OS was performed using the Kaplan-Meier method with corresponding two-sided 95% CI using the Brookmeyer and Crowley method [18].

## Results

### Patient presentation

We collected data from 26 treatment-naïve patients who gained access to tagraxofusp through the NPP between September 2019 and August 2022. No information on response status was reported for six patients; hence, 20 patients were included in the efficacy population and 26 patients in the safety population. Baseline characteristics of patients for whom assessments were performed and results known are shown in Table 1. The overall median age was 68 years, and 86% of patients had a baseline Eastern Cooperative Oncology Group (ECOG) performance status (PS) score of 0 or 1. The majority of the 21 patients with BPDCN assessed at baseline had skin and bone marrow involvement at the outset of therapy (81% each), with 67% showing signs of lymph node involvement and 50% spleen involvement. Most patients had proven CD123 expression of tumor cells in skin (100%) or bone marrow (83%) at baseline. Median serum albumin levels were normal in patients prior to tagraxofusp administration. Disease-related cytopenias were observed in more than half of the patients (e.g., 69% thrombocytopenias, Table 1). The median time from diagnosis to first-line treatment with tagraxofusp was 1.5 months. The median number of administered tagraxofusp cycles was 3 (range 1–9).

**Table 1:** Baseline characteristics

Characteristic	Treatment-naïve Patients (*N* = 26)		
Median age, years (range)	68 (21–87)		
Gender, no. (%)			
Male	22 (85)		
Female	4 (15)		
ECOG performance status prior to start^a^, no. (%)	*N* = 21 assessed		
0	9 (43)		
1	9 (43)		
2	3 (14)		
Disease involvement^a^, no. (%)			
Skin	17/21 (81)		
Bone marrow	17/21 (81)		
Lymph node	14/21 (67)		
Spleen	10/20 (50)		
Blood	9/20 (45)		
Central nervous system^b^	3/14 (21)		
Bulky disease at diagnosis (any lesion > 7 cm single or multiple), no. (%)	5 (19)^b^		
Immunophenotyping, no./*N* (%)^a^	Skin	Bone Marrow	
CD123 positive	15/15 (100)	10/12 (83)	
CD4 positive	16/16 (100)	9/12 (75)	
CD56 positive	15/17 (88)	9/12 (75)	
TCL1 positive	6/7 (86)	3/4 (75)	
CD303 positive	2/3 (67)	2/3 (67)	
TCF4 positive	0/0 (0)	1/2 (50)	
CD34 positive	1/10 (10)	0/12 (0)	
CD3 positive	1/16 (6)	1/12 (8)	
CD19 positive	0/8 (0)	1/10 (10)	
CD11c positive	0/2 (0)	0/7 (0)	
CD14 positive	0/4 (0)	0/6 (0)	
MPO positive	0/12 (0)	0/11 (0)	
Median albumin level prior to TAG start, g/dL (range)	3.95 (0–5.0)		
Hematologic status prior to TAG start^a^			
Anemia, no. (%)	12 (46)		
Median hemoglobin, g/dL (range)	9.1 (7.4–12.3)^c^		
Neutropenia, no. (%)	8 (31)		
Median neutrophils, 10^9^/L (range)	0.58 (0.3–1.5)^c^		
Thrombocytopenia, no. (%)	18 (69)		
Median platelets, 10^9^/L (range)	70 (14–131)^b^		
Median time from diagnosis to TAG start, months (range)	1.5 (0.4-9)^c^		

^a^ Results represent outcomes only in patients in whom test was performed and results known

^b^ As assessed and quantified by lumbar puncture with subsequent flow cytometry, as well as by MRI

ECOG, Eastern Cooperative Oncology Group; no., number; TAG, tagraxofusp

### Safety

Thirteen patients (50%) had a total of 19 CLS events diagnosed (Table 2). The most common symptoms associated with CLS events included weight gain (77%), edema (77%), hypotension (54%), and hypoalbuminemia (31%). The median time to occurrence of CLS was two days, with a median event duration of six days. The maximum grade of a CLS event was 2 in 54% of patients, 3 in 39% of patients, and 4 in 8% of patients. Interruption of tagraxofusp (42%) was the most common action taken for CLS events; intravenous albumin was administered for 17 CLS events (90%).

**Table 2:** Incidence and management of CLS events^a^ (all cycles)

	Treatment-naïve Patients (*N* = 26)
Patients with ≥ 1 formally diagnosed CLS event, no. (%)	13 (50)^b^
Patients with CLS events by maximum grade^c^, no. (%)	
1	0
2	7 (54)
3	5 (39)
4	1 (8)
Action taken on TAG, no. of events (%)	*N* = 19
No modification	5 (26)
Dose reduced	0
Drug interrupted	8 (42)
Drug withdrawn	5 (26)
Median time to CLS event,^d^ days (range)	2 (1–9)
Median duration of CLS events, days (range)	6 (1–37)

^a^ As reported by the investigator

^b^ Symptoms associated with CLS events were edema (*n* = 10 patients), weight gain (*n* = 10), hypotension (*n* = 7), hypoalbuminemia (*n* = 4), and other (*n* = 6)

^c^ Highest observed grade CLS event per patient

^d^ Calculated as CLS start date minus the tagraxofusp start date of the cycle in which the CLS event occurred. Time to CLS event was not calculated if the CLS event occurred after the tagraxofusp period

CLS, capillary leak syndrome; no., number; TAG, tagraxofusp

Most diagnosed CLS events (68%) occurred during cycle 1 (13 CLS events in 13 patients) while events in later cycles were less common. Four out of 13 patients had multiple CLS events (two patients had two events, and two patients had three events). No grade 4 CLS events occurred beyond cycle 1, and no grade 3 CLS events occurred beyond cycle 2.

Twelve (46%) patients had a total of 16 non-hematologic grade 3–4 AEs or SAEs deemed related to tagraxofusp, with a median time to resolution of 10.5 days (range 2–23) (Table 3). The most common treatment-related AE was temporary liver-related toxicity in eight patients (38%), which resolved at a median of 14 days (range 6–23). Treatment-related SAEs occurred in three patients and included CLS (two patients) and acute kidney injury (one patient). All non-hematologic treatment-related grade 3–4 AEs or SAEs occurred during cycle 1, except in one patient (6%) with events during cycle 2.

**Table 3:** Adverse events related to tagraxofusp^a^

	Treatment-naïve Patients (*N* = 26)	
	Patients, no. (%)	Time to Resolution, median days (range)
Grade 3–4 Hematologic AEs^b^	9 (35)	6.5 (1–23)
Thrombocytopenia	7 (27)	8 (3–10)
Neutropenia	4 (15)	5 (3–23)
Anemia	2 (8)	3 (1–5)
Grade 3–4 AEs or SAE Non-hematologic^c^	12 (46)	10.5 (2–23)
Hepatic toxicity^d^	10 (38)	14 (6–23)
Acute kidney injury	1 (4)	8 (8–8)
Hypoalbuminemia	1 (4)	3 (3–3)

^a^ See Table 2 for CLS incidence details

^b^ Serious adverse events (SAEs) were not collected for hematologic AEs

^c^ Includes CLS

^d^ Hepatic toxicity comprised AEs and of transitory aspartate aminotransferase increase, alanine aminotransferase increase, hepatic cytolysis, hypertransaminasemia, liver toxicity, and hepatotoxicity, which were reported as terms for discrete abnormalities

AE, adverse event; SAE, serious adverse event

Twelve (46%) patients had a total of 27 hematologic AEs deemed related to tagraxofusp. 23% of patients had at least one grade 1–2 hematologic AE, and 35% had at least one grade 3–4 hematologic AE related to tagraxofusp (Table 3). The median time for resolution of treatment-related hematologic AEs was 8 days (range 1–23).

**Fig. 1:**
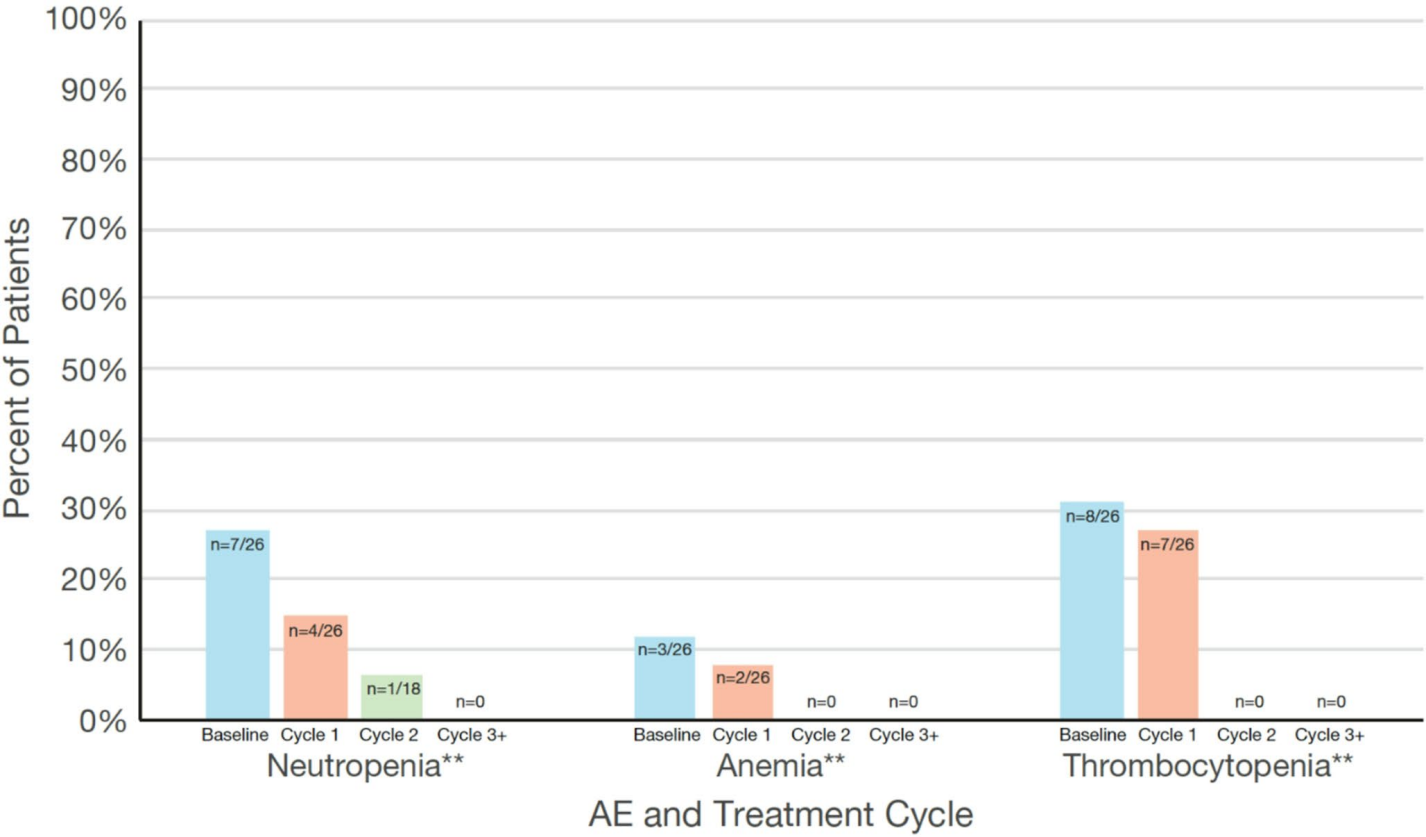
Grade 3–4* hematologic AEs at baseline and grade 3–4 treatment-related hematologic preferred term AEs over the course of tagraxofusp treatment. *Incidence of grade 3–4 treatment-related hematologic AEs was zero in cycles 3 and beyond. **Neutropenia, anemia, and thrombocytopenia are combined terms. AE, adverse event

Disease-related cytopenias prior to tagraxofusp included any grade of neutropenia, anemia, and thrombocytopenia and were present in 31%, 46%, and 69% of patients, respectively **(**Table 1**)**. During treatment with tagraxofusp, the grade 3–4, likely treatment-related, combined-term cytopenia-related AEs were reported mainly during cycle 1 (neutropenia count 15%; anemia 8%; thrombocytopenia 27%) and at lower frequencies with each subsequent cycle (cycle 2: 6% 0%, and 0%, respectively; none in cycle 3 and beyond). Of note, the incidence of these cytopenia-related combined-term AEs exactly matched the incidence of the preferred term cytopenia-related grade 3–4 AEs over time as shown in Fig. 1. The incidence of any grade of likely treatment-related combined-term cytopenia declined with each cycle of tagraxofusp: 46% in cycle 1, 28% in cycle 2, and 13% in cycle 3 and beyond.

Two patients died during their cycle 1 of tagraxofusp: one patient due to PD and one due to an infectious complication, possibly treatment-related. The latter patient suffered from a concomitant infection, grade 4 neutropenia and grade 4 thrombocytopenia at baseline, that did not improve during treatment with tagraxofusp.

### Efficacy

**Fig. 2:**
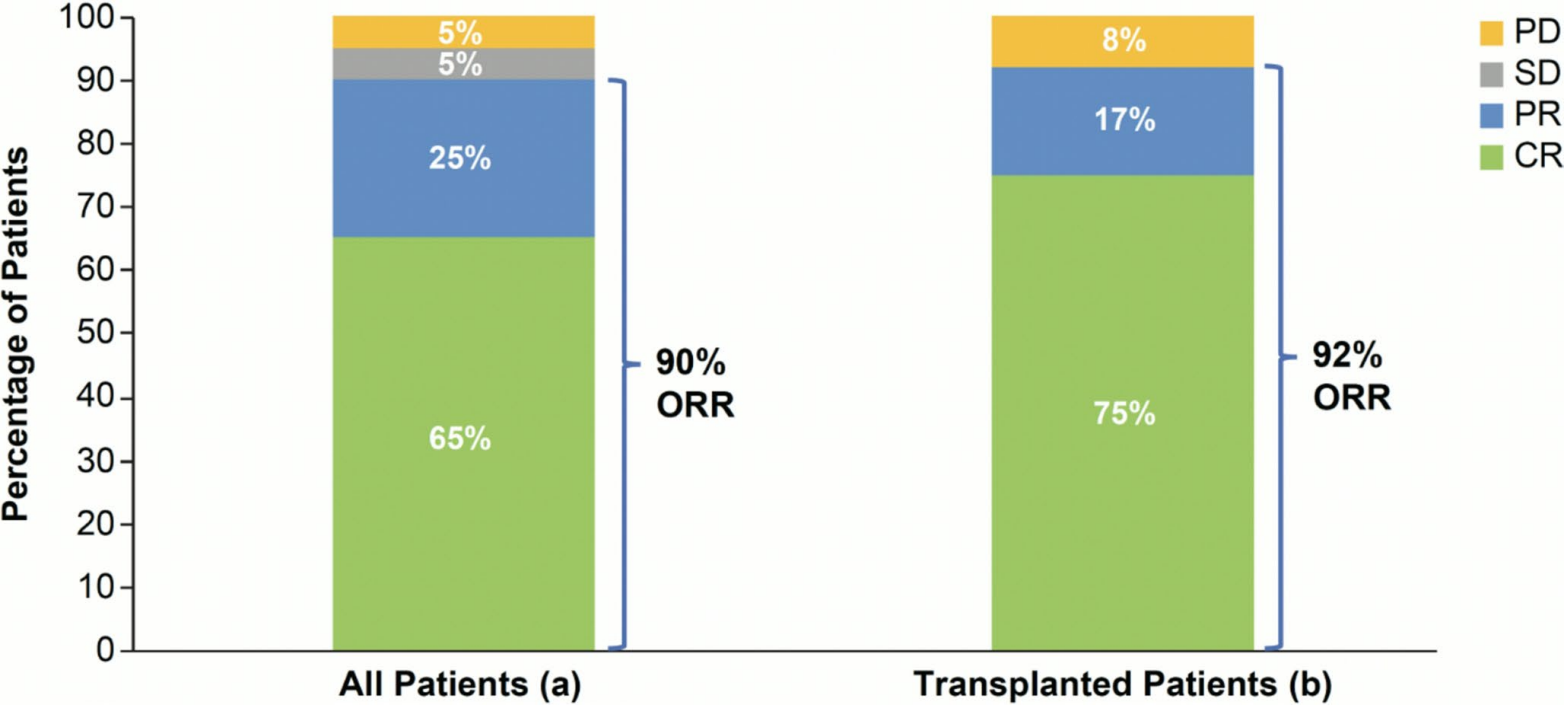
Best Overall Response (a) all response evaluable patients (*n* = 20) and (b) response evaluable transplanted patients (*n* = 12). CR, complete response; ORR, overall response rate; PD, progressive disease; PR, partial response; SD, stable disease

At a median follow-up of 13.5 months (range 0.07–49.6), the ORR was 90% (65% CR, 25% PR) in the 20 response-evaluable patients (Fig. 2). The median time to CR was 31.0 days (range 12–64), and the median time to first response was 23.5 days (range 1–75). The median DOR in responding patients was 10.3 months (95% CI 6.9–36.0), with a probability of continued response at 24 months of 40% (95% CI 0.1–0.6). The median DOR in the 13 patients with a CR was 11.4 months (95% CI 6.9–35.1), with a 40% probability of continued response at 24 months (95% CI 0.1–0.6).

Fifty percent (13/26) of all patients received an allogeneic HSCT during the study; no autologous HSCT was applied. Baseline characteristics of patients who underwent allogeneic HSCT and for whom assessments were performed included a median age of 64 years (range 21–72) and an ECOG PS score of 0–1 (90%); 82% and 64% had bone marrow and blood disease, respectively, at the start of therapy.

The median time to HSCT after the last tagraxofusp dose was 46 days (range 32–152). Fifty-five percent (11/20) of response-evaluable patients were bridged to transplantation after a response to tagraxofusp. One patient had no response to tagraxofusp, subsequently received Hyper-CVAD, and then proceeded to transplantation. Another transplanted patient was not evaluable for response before HSCT. Transplanted patients received a median of three cycles (range 1–7) of tagraxofusp before HSCT. Disease status prior to HSCT was CR in 54% and PR in 46%. At a median follow-up of 26 months (range 7.5–49.6), the ORR in the 12 response-evaluable patients who were bridged to HSCT was 92% (75% CR, 17% PR) (Fig. 2). Median time to tagraxofusp CR in transplanted patients was 29.0 days (range 12–59), and median time to response was 21.0 days (range 1–75). The median DOR in patients who were bridged to transplantation was 14.7 months (95% CI 3.2–not estimable [NE]), with a 40% probability of continued response at 24 months (95% CI 0.1–0.7). In the nine patients with CR after tagraxofusp, the median DOR was 14.1 months (95% CI 1.8–NE), with a 40% probability of continued response at 24 months (95% CI, 0.1–0.7). After HSCT, 46% of patients experienced a GvHD, which was acute in four and chronic in two patients. There were no SOS (VOD) events in two assessable patients for which data were reported. Disease status after HSCT was CR in 77%, PR in 8%, and PD/relapse in 15%.

**Fig. 3:**
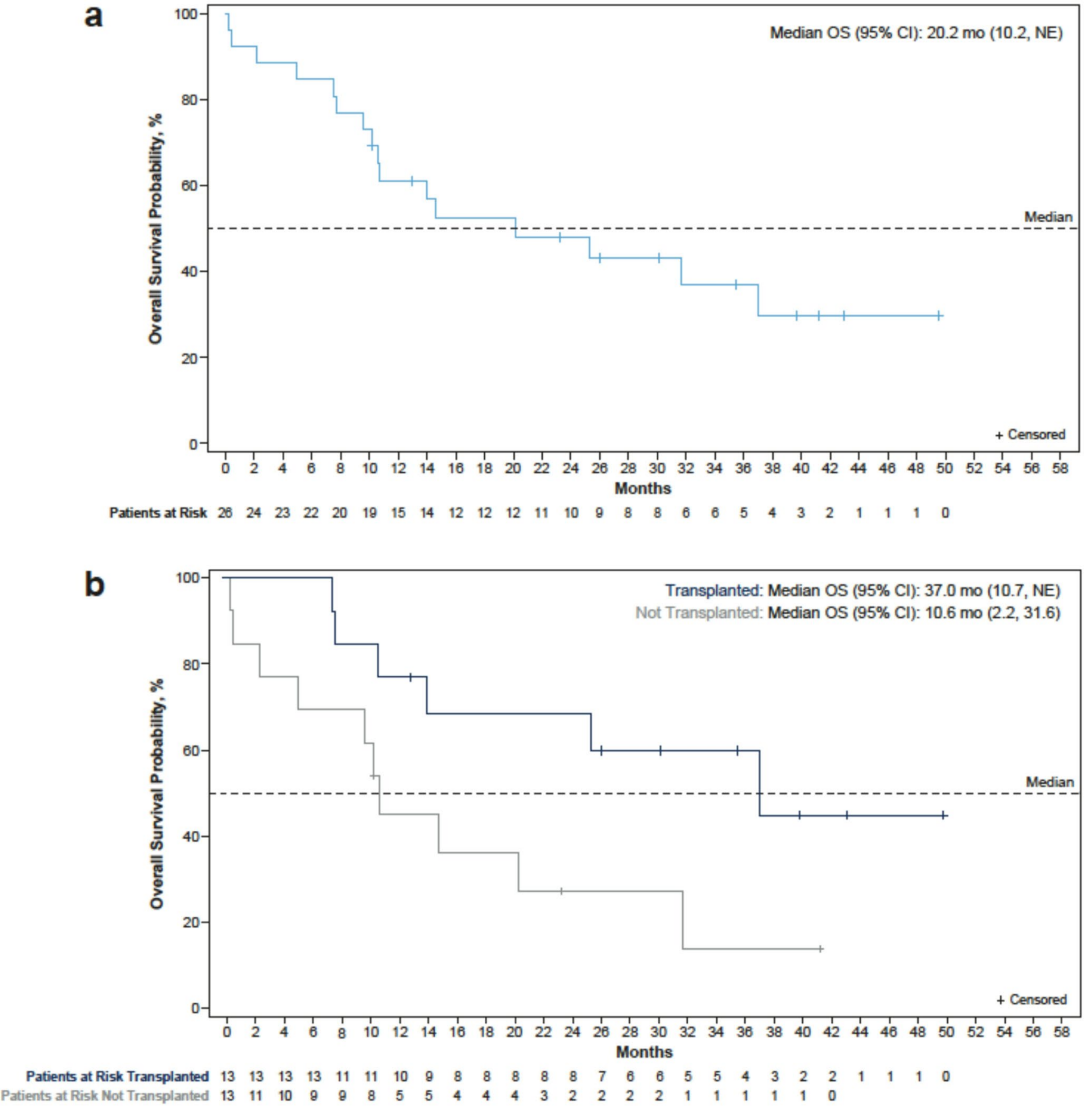
Overall Survival (OS) from time of TAG initiation for (a) All Treatment-Naïve Patients; and (b) Transplanted and Non-transplanted Treatment-Naïve Patients. CI, confidence interval; mo, months; NE, not estimable; OS, overall survival

In the overall population, the median PFS was 9.8 months (95% CI 5.5–14.7), and the median OS was 20.2 months (95% CI 10.2–NE; Fig. 3a). In the 13 patients who were bridged to HSCT (12 of whom were assessed and responding to tagraxofusp prior to HSCT), the median PFS was 7.9 months (95% CI 3.2–37.0), and the median OS was 37.0 months (95% CI 10.7–NE; Fig. 3b). For the 13 non-transplanted patients, the median PFS was 10.2 months (95% CI 1.2–14.7), and the median OS was 10.6 months (95% CI 2.2–31.6)(Fig. 3b). OS from time of diagnosis for both transplanted and non-transplanted patients is shown in Supplemental Fig. 1. At last reported follow-up, nine patients (35%) were alive (two with and seven without documented disease, respectively). Sixteen patients (62%) had died; reasons included PD (13 patients), other concurrent disease (1 patient), and infectious complication (2 patients). Status for one patient was missing.

Three patients (15% of the response-evaluable population and 12% of the total population) had documented CNS involvement at the start of tagraxofusp treatment, two of whom received intrathecal chemotherapy (ITC) before and after tagraxofusp initiation. Both patients achieved a CR in marrow and were bridged to transplantation. The third patient did not receive ITC and achieved a PR with tagraxofusp.

In addition, eight patients (31%) without documented CNS involvement at baseline received ITC treatment. Five patients were treated with ITC (four prophylactically) before initiating tagraxofusp; three of whom also received ITC after tagraxofusp initiation. Response to tagraxofusp was evaluable in four of these patients, three achieved a CR as BOR and one achieved a PR. Lastly, ITC was administered to three patients (two prophylactically) after initiation of tagraxofusp. Response to tagraxofusp was evaluable in three of these patients, both of whom achieved a CR as BOR.

## Discussion

The European NPP that commenced in 2019 enabled patients with BPDCN an opportunity to receive tagraxofusp as the first targeted drug in this aggressive orphan disease, ahead of commercial availability. Valuable insights for everyday clinical practice, beyond the clinical trial evidence [7, 8], were garnered from these retrospective data of treatment-naïve patients who received tagraxofusp in a real-world setting. Overall, this study further establishes tagraxofusp as the first-line treatment of choice for a broad spectrum of patients with BPDCN.

This study population mirrored the typical BPDCN patient profile [4, 5, 12, 13, 17, 19, 20]: a higher incidence in males (85%), age ≥ 60 years (median of 68 years), and hallmark BPDCN features, with 81% skin, 81% bone marrow, 67% lymph node, and 21% CNS disease at the time of first-line treatment. Moreover, CD123 expression of tumor cells in skin and bone marrow biopsies (in patients with available immunophenotyping) was detected in 100% and 83% of patients, respectively.

Historical studies that evaluated multi-agent chemotherapy regimens for treatment-naïve patients with BPDCN reported CR rates ranging from 41% to 69% and median OS ranging from 6 to 19 months [5, 13–15, 21, 22]. These heterogeneous and retrospective cohort studies included a range of treatments and lacked uniform, prespecified, multisystem response criteria, warranting caution in their interpretation. Although more recent retrospective studies evaluating front-line, hyper-fractionated cyclophosphamide, vincristine, doxorubicin, and dexamethasone (Hyper-CVAD)-based regimens, combined with high-dose methotrexate and cytarabine, demonstrated high CR rates and OS, patients were younger than the usual BPDCN population, adding an element of intrinsic confounding and making these results difficult to generalize [23, 24]. Indeed, in a report from an ongoing prospective study of chemotherapy for BPDCN in France, 38% of patients were not able to tolerate the planned treatment, and only 38% of the cohort could be bridged to transplantation [25]. Despite the challenges of historical evidence and absence of robust prospective evaluation of polychemotherapy, our findings with tagraxofusp monotherapy, including the 20.2-months median OS, exceed historical efficacy outcomes reported with multi-agent chemotherapy [12].

In this real-world analysis, tagraxofusp monotherapy was associated with an encouraging 65% CR rate and 90% ORR, in line with the 57% CR rate and 75% ORR observed in the pivotal trial [7, 8]. Although the current cohort of patients appears to have had more overt disease (as evidenced by disease-related cytopenias and higher rates of involvement of bone marrow, lymph nodes, blood, spleen, and CNS) than patients in the pivotal clinical trial, responses were rapid (time to response 23.5 and 31 days for overall response and CR, respectively) and durable. The median DOR (10.3 months) in this analysis might have been attributed to the more difficult-to-treat patient population relative to patients in the prospective trial (median DOR 24.9 months). In addition, within clinical trials, a standardized approach as well as fixed cadence of assessment to capture responses is employed. However, in a real-world setting, this can be challenging and increase heterogeneity in the data, which could partly explain these differences in DOR. However, the 20.2-month median OS in this real-world population is supportive of that reported in the pivotal trial (15.8 months) [8]. Moreover, 50% of patients (with any response) in our analysis were bridged to HSCT, confirming the effectiveness of tagraxofusp to bridge patients to HSCT observed in the pivotal trial [8].

Importantly, the safety analysis confirms that tagraxofusp is not myelosuppressive in a real-world setting. Despite notable baseline (disease-related) cytopenias, the incidence of grade 3–4 treatment-related thrombocytopenia, neutropenia, and anemia during tagraxofusp monotherapy were lower at 27%, 15%, and 8%, respectively. The majority of grade 3–4 treatment-related AEs occurred during cycle 1 and resolved quickly. In fact, the incidence of hematologic treatment-related AEs decreased notably with each subsequent cycle of tagraxofusp. This is a major advantage over conventional cytostatic protocols with rather cumulative hematologic toxicities in a disease that is intrinsically associated with coexisting myelodysplastic or myeloproliferative pathologies. Therefore, it was also encouraging to see improvements in hematologic parameters upon tagraxofusp initiation in patients who had considerable cytopenias at baseline.

In addition, no new safety issues were observed with tagraxofusp monotherapy in this real-world setting. CLS, an AE of special interest, was mostly limited to the first two cycles (84%). CLS management guidelines that included training on CLS assessment and monitoring for all involved physicians, nurses, and pharmacists appear to have been effective as most CLS events resolved (95%). Transitory liver-related toxicity (that included elevation of liver transaminases) fulfilling grade 3–4 criteria, as observed here in 38%, were less frequent than previously reported [8].

Contrary to chemotherapy, whose toxicity profile, in particular neutropenia-associated infections, can preclude a successful transition to HSCT, our data demonstrate the feasibility and success of tagraxofusp in bridging patients to a HSCT. Half of the patients in this analysis were bridged to allogeneic HSCT. In patients bridged to HSCT, responses to tagraxofusp were robust and durable (75% CR, 14.7-months DOR), and the median OS was nearly doubled (37 months). As expected, the transplanted patients were younger than those who did not undergo HSCT (median age 64 years vs. 72 years; ORR 92% vs. 88%). Overall, while acknowledging the pitfalls of indirect comparisons given the more favorable toxicity profile of tagraxofusp over intensive induction polychemotherapy [13, 24–26], tagraxofusp offers an efficacious and well-tolerated targeted treatment strategy for a broad spectrum of treatment-naïve patients with BPDCN, also allowing patients who might not have tolerated polychemotherapy to bridge to the potentially curative HSCT.

Although real-world studies, such as this report, are limited by retrospective and sometimes fragmented or redundant data collection, the findings of this analysis from a representative BPDCN cohort corroborate the known safety and efficacy of tagraxofusp and reveal its applicability to a broad range of patients with BPDCN, including those with poor performance status. The consistency of these results with those of the prospective, pivotal trial substantiates our real-world findings. Taken together, the outcomes of the current study support tagraxofusp monotherapy as the standard of care in treatment-naïve patients with BPDCN.

## Conclusions

With more than two years of follow-up, patients with BPDCN who received tagraxofusp monotherapy as first-line treatment in the real world achieved fast, durable responses and prolonged survival, regardless of initial risk features such as cytopenias or CNS involvement. Tagraxofusp demonstrated a manageable, non-cumulative safety profile, with no new safety issues. With proper patient selection, monitoring for early recognition and directed intervention, CLS associated with tagraxofusp is manageable and mostly limited to the first two cycles and predominantly mild to moderate. These real-world results confirm tagraxofusp as a first-line standard of care for most patients with treatment-naïve BPDCN and an effective and safe option to bridge eligible patients to transplantation.

## Electronic supplementary material

Below is the link to the electronic supplementary material.

Supplementary Material 1

## Data Availability

Given the retrospective nature of the study, we are unable to share individual patient data. However, aggregated data may be provided upon specific request.
