# Supplementary material for: Durable outcomes with manageable safety leading to prolonged survival with tagraxofusp for treatment-naïve patients with blastic plasmacytoid dendritic cell neoplasm: real world results from a European Named Patient Program
Source: Ann Hematol. 2025 Aug 2;104(8):4121–31. doi: 10.1007/s00277-025-06493-w (PMC12432076; doi:10.1007/s00277-025-06493-w)
Supplement: Supplementary file 1 — Supplementary Material 1 [file 277_2025_6493_MOESM1_ESM.docx]

**Supplemental Fig. 1** Overall Survival (OS) for Transplanted and Non-transplanted Treatment-Naïve Patients from Time of Diagnosis


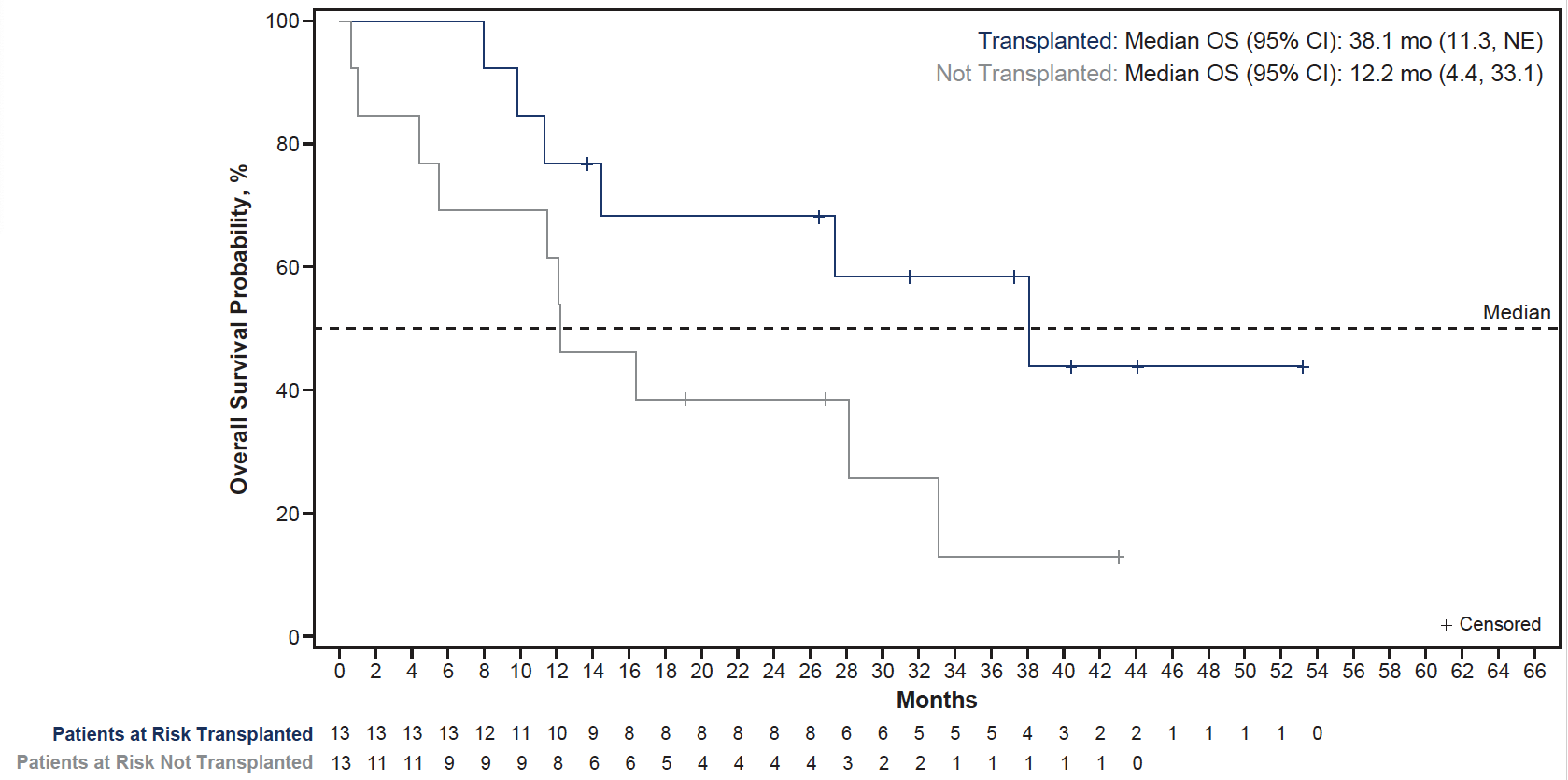


CI, confidence interval; mo, months; NE, not estimable; OS, overall survival.
